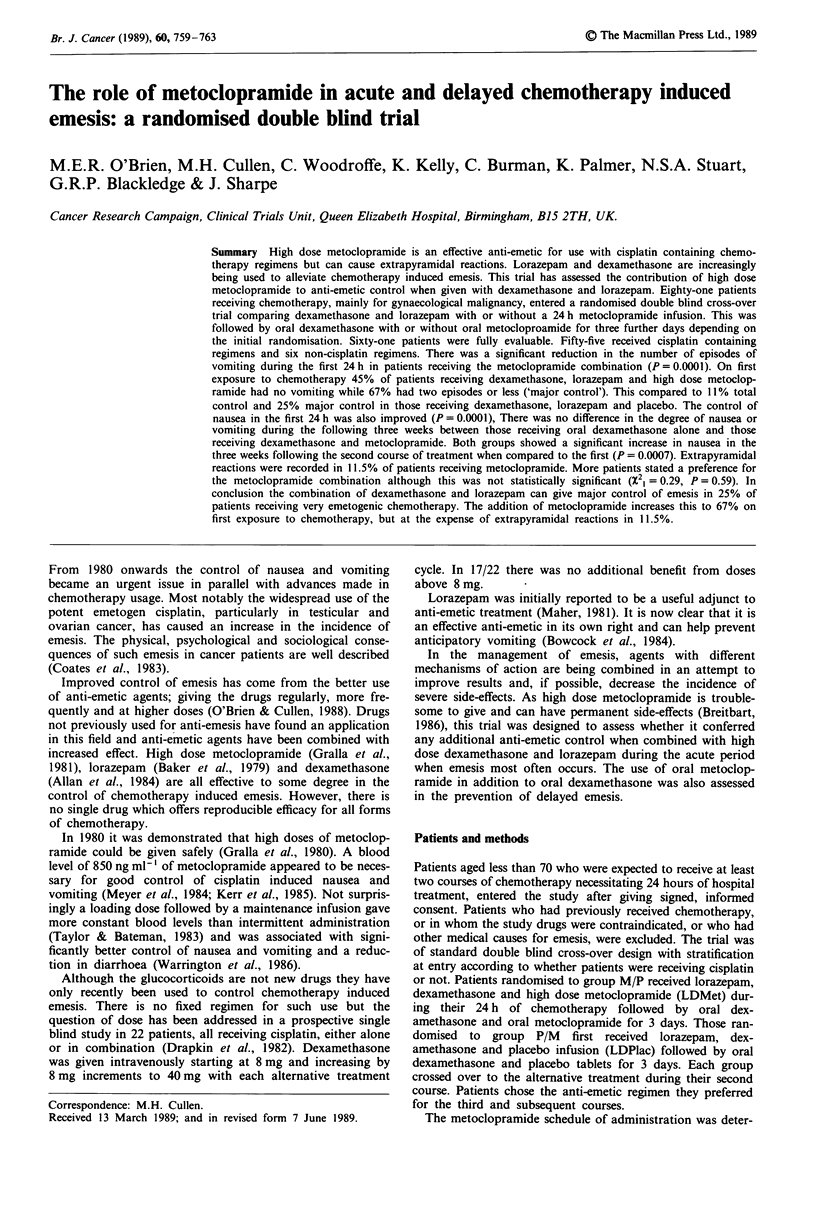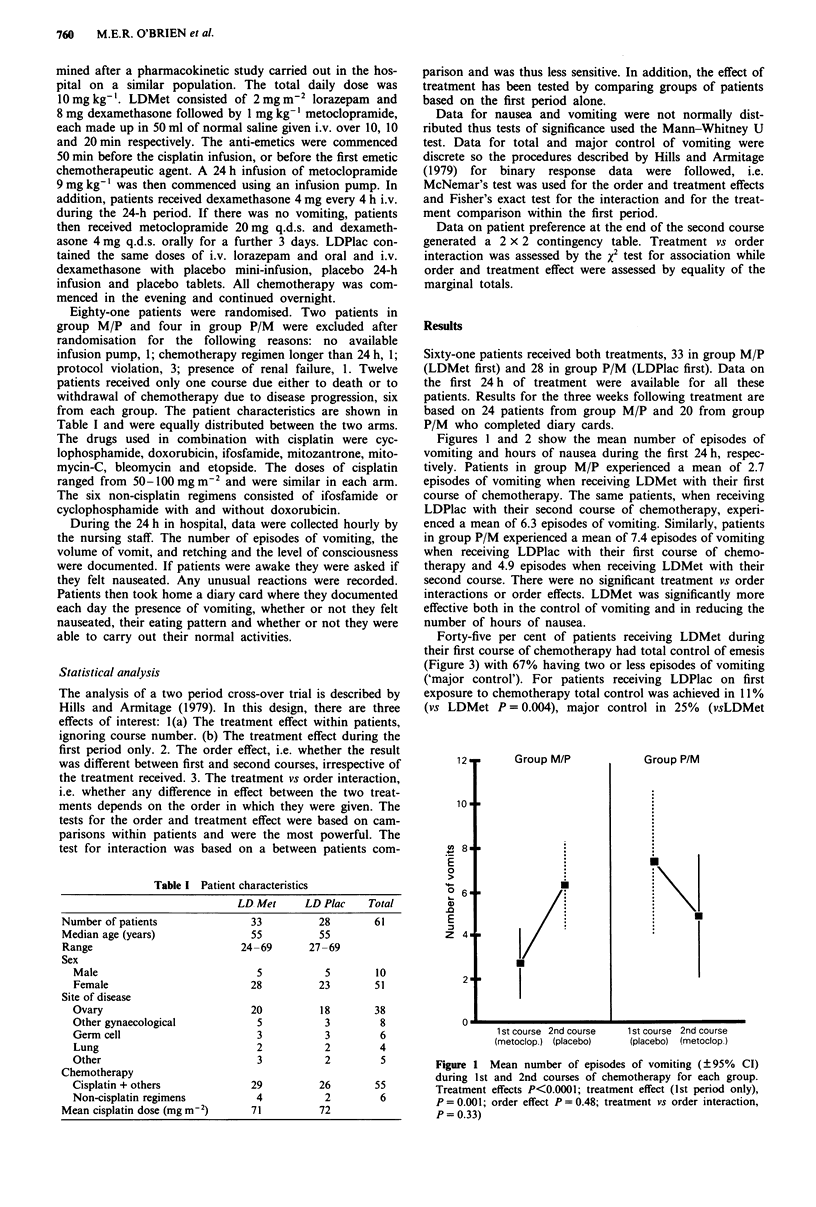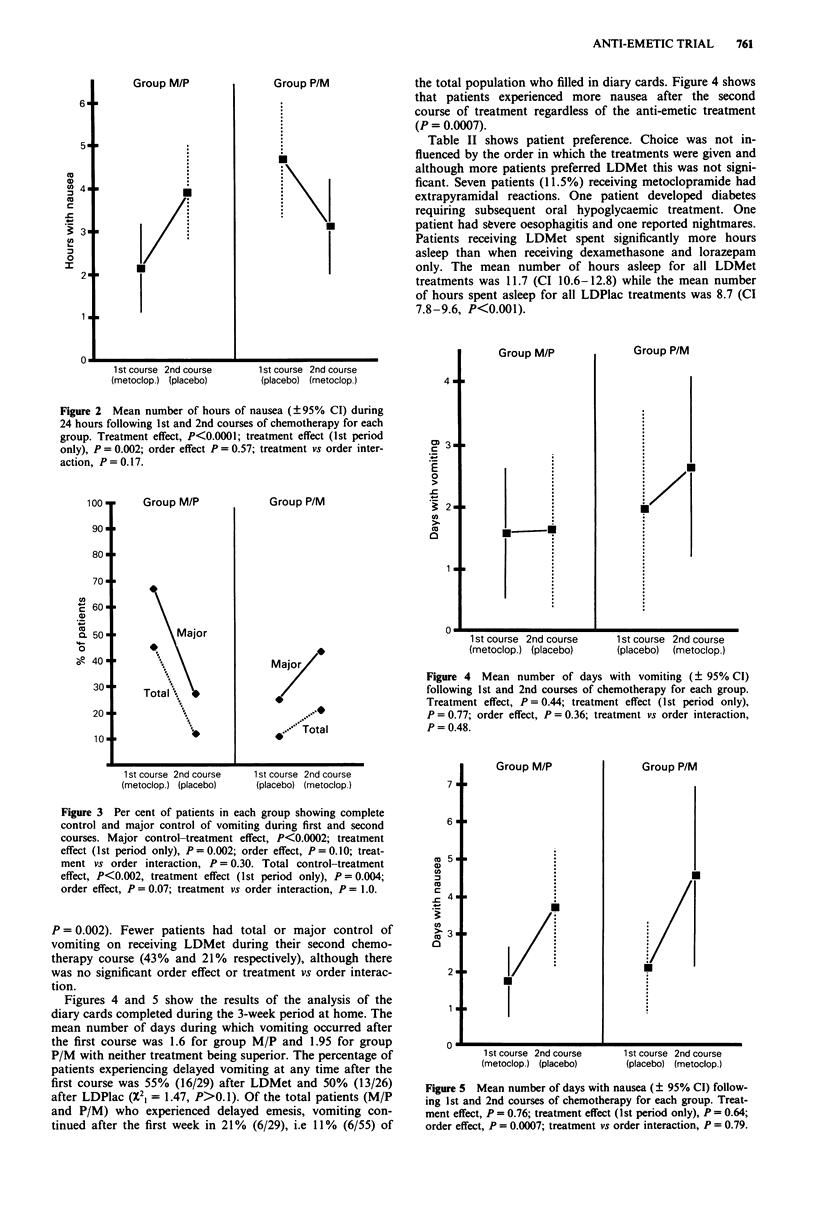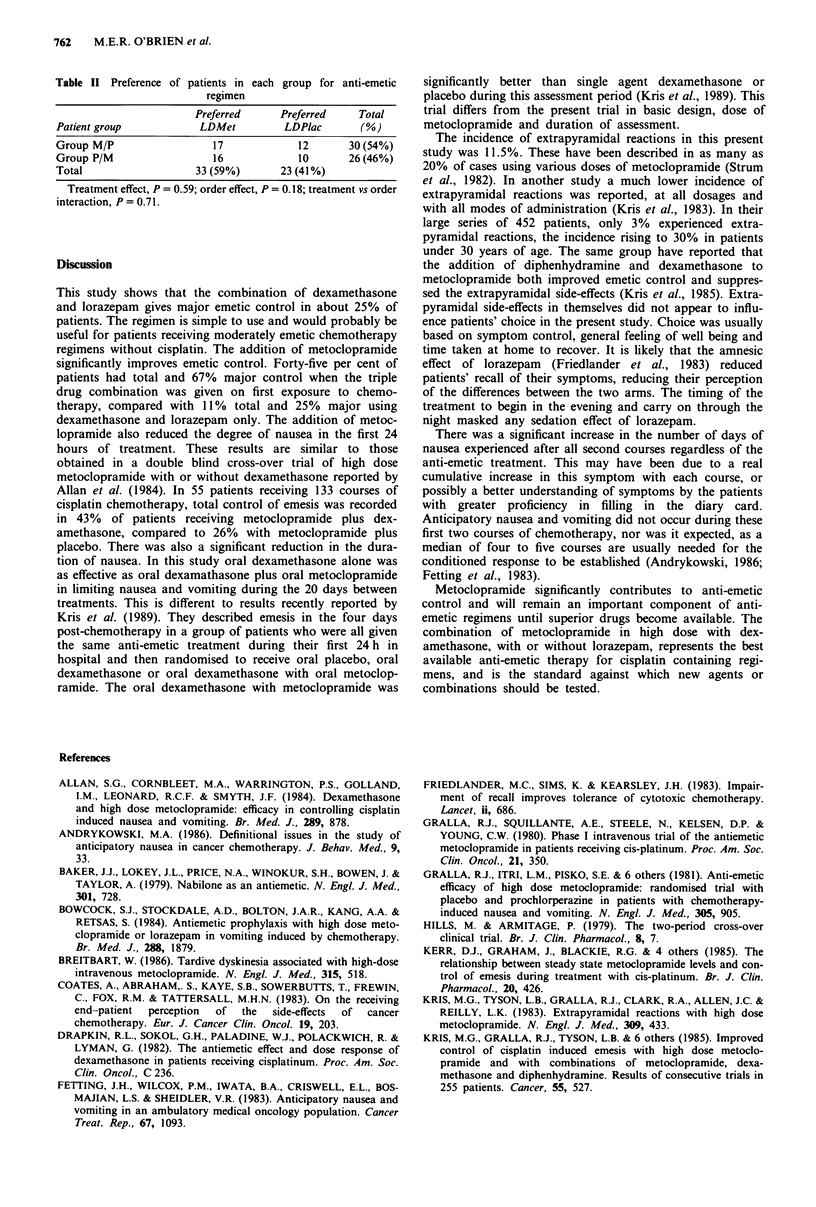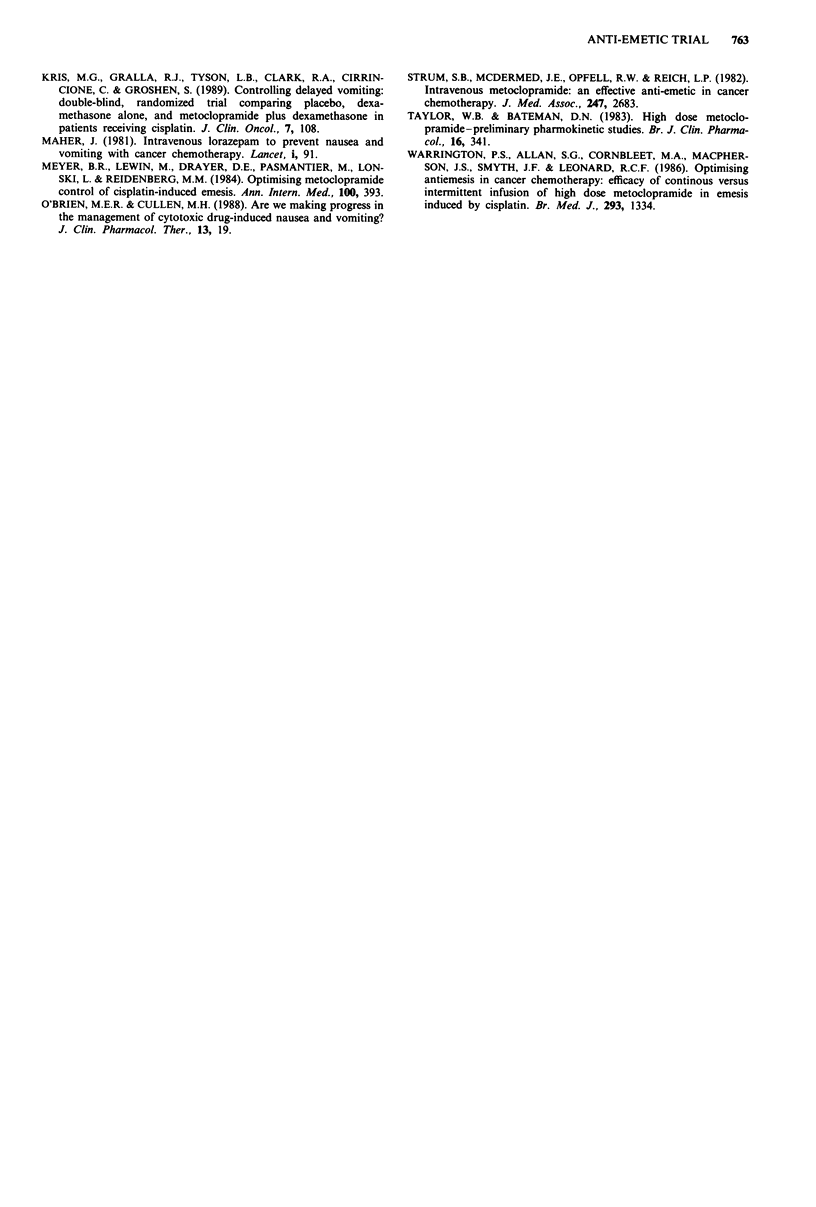# The role of metoclopramide in acute and delayed chemotherapy induced emesis: a randomised double blind trial.

**DOI:** 10.1038/bjc.1989.354

**Published:** 1989-11

**Authors:** M. E. O'Brien, M. H. Cullen, C. Woodroffe, K. Kelly, C. Burman, K. Palmer, N. S. Stuart, G. R. Blackledge, J. Sharpe

**Affiliations:** Cancer Research Campaign, Clinical Trials Unit, Queen Elizabeth Hospital, Birmingham, UK.

## Abstract

High dose metoclopramide is an effective anti-emetic for use with cisplatin containing chemotherapy regimens but can cause extrapyramidal reactions. Lorazepam and dexamethasone are increasingly being used to alleviate chemotherapy induced emesis. This trial has assessed the contribution of high dose metoclopramide to anti-emetic control when given with dexamethasone and lorazepam. Eight-one patients receiving chemotherapy, mainly for gynaecological malignancy, entered a randomised double blind cross-over trial comparing dexamethasone and lorazepam with or without a 24 h metoclopramide infusion. This was followed by oral dexamethasone with or without oral metoclopramide for three further days depending on the initial randomisation. Sixty-one patients were fully evaluable. Fifty-five received cisplatin containing regimens and six non-cisplatin regimens. There was a significant reduction in the number of episodes of vomiting during the first 24 h in patients receiving the metoclopramide combination (P = 0.0001). On first exposure to chemotherapy 45% of patients receiving dexamethasone, lorazepam and high dose metoclopramide had no vomiting while 67% had two episodes or less ('major control'). This compared to 11% total control and 25% major control in those receiving dexamethasone, lorazepam and placebo. The control of nausea in the first 24 h was also improved (P = 0.0001). There was no difference in the degree of nausea or vomiting during the following three weeks between those receiving oral dexamethasone alone and those receiving dexamethasone and metoclopramide. Both groups showed a significant increase in nausea in the three weeks following the second course of treatment when compared to the first (P = 0.0007). Extrapyramidal reactions were recorded in 11.5% of patients receiving metoclopramide. More patients stated a preference for the metoclopramide combination although this was not statistically significant (chi 2(1) = 0.29, P = 0.59). In conclusion the combination of dexamethasone and lorazepam can give major control of emesis in 25% of patients receiving very emetogenic chemotherapy. The addition of metoclopramide increases this to 67% on first exposure to chemotherapy, but at the expense of extrapyramidal reactions in 11.5%.


Br. J. Cancer (1989), 60, 759–763
© The Macmillan Press Ltd., 1989

### The role of metoclopramide in acute and delayed chemotherapy induced emesis: a randomised double blind trial

M.E.R. O'Brien, M.H. Cullen, C. Woodroffe, K. Kelly, C. Burman, K. Palmer, N.S.A. Stuart, G.R.P. Blackledge & J. Sharpe

*Cancer Research Campaign, Clinical Trials Unit, Queen Elizabeth Hospital, Birmingham, B15 2TH, UK.*

**Summary** High dose metoclopramide is an effective anti-emetic for use with cisplatin containing chemotherapy regimens but can cause extrapyramidal reactions. Lorazepam and dexamethasone are increasingly being used to alleviate chemotherapy induced emesis. This trial has assessed the contribution of high dose metoclopramide to anti-emetic control when given with dexamethasone and lorazepam. Eighty-one patients receiving chemotherapy, mainly for gynaecological malignancy, entered a randomised double blind cross-over trial comparing dexamethasone and lorazepam with or without a 24 h metoclopramide infusion. This was followed by oral dexamethasone with or without oral metocloproamide for three further days depending on the initial randomisation. Sixty-one patients were fully evaluable. Fifty-five received cisplatin containing regimens and six non-cisplatin regimens. There was a significant reduction in the number of episodes of vomiting during the first 24 h in patients receiving the metoclopramide combination ($P = 0.0001$). On first exposure to chemotherapy 45% of patients receiving dexamethasone, lorazepam and high dose metoclopramide had no vomiting while 67% had two episodes or less ('major control'). This compared to 11% total control and 25% major control in those receiving dexamethasone, lorazepam and placebo. The control of nausea in the first 24 h was also improved ($P = 0.0001$), There was no difference in the degree of nausea or vomiting during the following three weeks between those receiving oral dexamethasone alone and those receiving dexamethasone and metoclopramide. Both groups showed a significant increase in nausea in the three weeks following the second course of treatment when compared to the first ($P = 0.0007$). Extrapyramidal reactions were recorded in 11.5% of patients receiving metoclopramide. More patients stated a preference for the metoclopramide combination although this was not statistically significant ($\chi^2_1 = 0.29$, $P = 0.59$). In conclusion the combination of dexamethasone and lorazepam can give major control of emesis in 25% of patients receiving very emetogenic chemotherapy. The addition of metoclopramide increases this to 67% on first exposure to chemotherapy, but at the expense of extrapyramidal reactions in 11.5%.

From 1980 onwards the control of nausea and vomiting became an urgent issue in parallel with advances made in chemotherapy usage. Most notably the widespread use of the potent emetogen cisplatin, particularly in testicular and ovarian cancer, has caused an increase in the incidence of emesis. The physical, psychological and sociological consequences of such emesis in cancer patients are well described (Coates *et al.*, 1983).

Improved control of emesis has come from the better use of anti-emetic agents; giving the drugs regularly, more frequently and at higher doses (O'Brien & Cullen, 1988). Drugs not previously used for anti-emesis have found an application in this field and anti-emetic agents have been combined with increased effect. High dose metoclopramide (Gralla *et al.*, 1981), lorazepam (Baker *et al.*, 1979) and dexamethasone (Allan *et al.*, 1984) are all effective to some degree in the control of chemotherapy induced emesis. However, there is no single drug which offers reproducible efficacy for all forms of chemotherapy.

In 1980 it was demonstrated that high doses of metoclopramide could be given safely (Gralla *et al.*, 1980). A blood level of 850 ng ml$^{-1}$ of metoclopramide appeared to be necessary for good control of cisplatin induced nausea and vomiting (Meyer *et al.*, 1984; Kerr *et al.*, 1985). Not surprisingly a loading dose followed by a maintenance infusion gave more constant blood levels than intermittent administration (Taylor & Bateman, 1983) and was associated with significantly better control of nausea and vomiting and a reduction in diarrhoea (Warrington *et al.*, 1986).

Although the glucocorticoids are not new drugs they have only recently been used to control chemotherapy induced emesis. There is no fixed regimen for such use but the question of dose has been addressed in a prospective single blind study in 22 patients, all receiving cisplatin, either alone or in combination (Drapkin *et al.*, 1982). Dexamethasone was given intravenously starting at 8 mg and increasing by 8 mg increments to 40 mg with each alternative treatment cycle. In 17/22 there was no additional benefit from doses above 8 mg.

Correspondence: M.H. Cullen.
Received 13 March 1989; and in revised form 7 June 1989.

Lorazepam was initially reported to be a useful adjunct to anti-emetic treatment (Maher, 1981). It is now clear that it is an effective anti-emetic in its own right and can help prevent anticipatory vomiting (Bowcock *et al.*, 1984).

In the management of emesis, agents with different mechanisms of action are being combined in an attempt to improve results and, if possible, decrease the incidence of severe side-effects. As high dose metoclopramide is troublesome to give and can have permanent side-effects (Breitbart, 1986), this trial was designed to assess whether it conferred any additional anti-emetic control when combined with high dose dexamethasone and lorazepam during the acute period when emesis most often occurs. The use of oral metoclopramide in addition to oral dexamethasone was also assessed in the prevention of delayed emesis.

### Patients and methods

Patients aged less than 70 who were expected to receive at least two courses of chemotherapy necessitating 24 hours of hospital treatment, entered the study after giving signed, informed consent. Patients who had previously received chemotherapy, or in whom the study drugs were contraindicated, or who had other medical causes for emesis, were excluded. The trial was of standard double blind cross-over design with stratification at entry according to whether patients were receiving cisplatin or not. Patients randomised to group M/P received lorazepam, dexamethasone and high dose metoclopramide (LDMet) during their 24 h of chemotherapy followed by oral dexamethasone and oral metoclopramide for 3 days. Those randomised to group P/M first received lorazepam, dexamethasone and placebo infusion (LDPlac) followed by oral dexamethasone and placebo tablets for 3 days. Each group crossed over to the alternative treatment during their second course. Patients chose the anti-emetic regimen they preferred for the third and subsequent courses.

The metoclopramide schedule of administration was determined after a pharmacokinetic study carried out in the hospital on a similar population. The total daily dose was 10 mg kg$^{-1}$. LDMet consisted of 2 mg m$^{-2}$ lorazepam and 8 mg dexamethasone followed by 1 mg kg$^{-1}$ metoclopramide, each made up in 50 ml of normal saline given i.v. over 10, 10 and 20 min respectively. The anti-emetics were commenced 50 min before the cisplatin infusion, or before the first emetic chemotherapeutic agent. A 24 h infusion of metoclopramide 9 mg kg$^{-1}$ was then commenced using an infusion pump. In addition, patients received dexamethasone 4 mg every 4 h i.v. during the 24-h period. If there was no vomiting, patients then received metoclopramide 20 mg q.d.s. and dexamethasone 4 mg q.d.s. orally for a further 3 days. LDPlac contained the same doses of i.v. lorazepam and oral and i.v. dexamethasone with placebo mini-infusion, placebo 24-h infusion and placebo tablets. All chemotherapy was commenced in the evening and continued overnight.

Eighty-one patients were randomised. Two patients in group M/P and four in group P/M were excluded after randomisation for the following reasons: no available infusion pump, 1; chemotherapy regimen longer than 24 h, 1; protocol violation, 3; presence of renal failure, 1. Twelve patients received only one course due either to death or to withdrawal of chemotherapy due to disease progression, six from each group. The patient characteristics are shown in Table I and were equally distributed between the two arms. The drugs used in combination with cisplatin were cyclophosphamide, doxorubicin, ifosfamide, mitozantrone, mitomycin-C, bleomycin and etopside. The doses of cisplatin ranged from 50–100 mg m$^{-2}$ and were similar in each arm. The six non-cisplatin regimens consisted of ifosfamide or cyclophosphamide with and without doxorubicin.

During the 24 h in hospital, data were collected hourly by the nursing staff. The number of episodes of vomiting, the volume of vomit, and retching and the level of consciousness were documented. If patients were awake they were asked if they felt nauseated. Any unusual reactions were recorded. Patients then took home a diary card where they documented each day the presence of vomiting, whether or not they felt nauseated, their eating pattern and whether or not they were able to carry out their normal activities.

### *Statistical analysis*

The analysis of a two period cross-over trial is described by Hills and Armitage (1979). In this design, there are three effects of interest: 1(a) The treatment effect within patients, ignoring course number. (b) The treatment effect during the first period only. 2. The order effect, i.e. whether the result was different between first and second courses, irrespective of the treatment received. 3. The treatment *vs* order interaction, i.e. whether any difference in effect between the two treatments depends on the order in which they were given. The tests for the order and treatment effect were based on camparisons within patients and were the most powerful. The test for interaction was based on a between patients comparison and was thus less sensitive. In addition, the effect of treatment has been tested by comparing groups of patients based on the first period alone.

Data for nausea and vomiting were not normally distributed thus tests of significance used the Mann–Whitney U test. Data for total and major control of vomiting were discrete so the procedures described by Hills and Armitage (1979) for binary response data were followed, i.e. McNemar's test was used for the order and treatment effects and Fisher's exact test for the interaction and for the treatment comparison within the first period.

Data on patient preference at the end of the second course generated a 2 × 2 contingency table. Treatment *vs* order interaction was assessed by the $\chi^2$ test for association while order and treatment effect were assessed by equality of the marginal totals.

**Table I** Patient characteristics

| | *LD Met* | *LD Plac* | *Total* |
|---|---|---|---|
| Number of patients | 33 | 28 | 61 |
| Median age (years) | 55 | 55 | |
| Range | 24–69 | 27–69 | |
| Sex | | | |
| Male | 5 | 5 | 10 |
| Female | 28 | 23 | 51 |
| Site of disease | | | |
| Ovary | 20 | 18 | 38 |
| Other gynaecological | 5 | 3 | 8 |
| Germ cell | 3 | 3 | 6 |
| Lung | 2 | 2 | 4 |
| Other | 3 | 2 | 5 |
| Chemotherapy | | | |
| Cisplatin + others | 29 | 26 | 55 |
| Non-cisplatin regimens | 4 | 2 | 6 |
| Mean cisplatin dose (mg m$^{-2}$) | 71 | 72 | |

### Results

Sixty-one patients received both treatments, 33 in group M/P (LDMet first) and 28 in group P/M (LDPlac first). Data on the first 24 h of treatment were available for all these patients. Results for the three weeks following treatment are based on 24 patients from group M/P and 20 from group P/M who completed diary cards.

Figures 1 and 2 show the mean number of episodes of vomiting and hours of nausea during the first 24 h, respectively. Patients in group M/P experienced a mean of 2.7 episodes of vomiting when receiving LDMet with their first course of chemotherapy. The same patients, when receiving LDPlac with their second course of chemotherapy, experienced a mean of 6.3 episodes of vomiting. Similarly, patients in group P/M experienced a mean of 7.4 episodes of vomiting when receiving LDPlac with their first course of chemotherapy and 4.9 episodes when receiving LDMet with their second course. There were no significant treatment *vs* order interactions or order effects. LDMet was significantly more effective both in the control of vomiting and in reducing the number of hours of nausea.

Forty-five per cent of patients receiving LDMet during their first course of chemotherapy had total control of emesis (Figure 3) with 67% having two or less episodes of vomiting ('major control'). For patients receiving LDPlac on first exposure to chemotherapy total control was achieved in 11% (*vs* LDMet $P = 0.004$), major control in 25% (*vs*LDMet

**Figure 1** Mean number of episodes of vomiting (±95% CI) during 1st and 2nd courses of chemotherapy for each group. Treatment effects $P<0.0001$; treatment effect (1st period only), $P = 0.001$; order effect $P = 0.48$; treatment *vs* order interaction, $P = 0.33$)

**Figure 2** Mean number of hours of nausea (±95% CI) during 24 hours following 1st and 2nd courses of chemotherapy for each group. Treatment effect, $P<0.0001$; treatment effect (1st period only), $P = 0.002$; order effect $P = 0.57$; treatment *vs* order interaction, $P = 0.17$.

**Figure 3** Per cent of patients in each group showing complete control and major control of vomiting during first and second courses. Major control–treatment effect, $P<0.0002$; treatment effect (1st period only), $P = 0.002$; order effect, $P = 0.10$; treatment *vs* order interaction, $P = 0.30$. Total control–treatment effect, $P<0.002$, treatment effect (1st period only), $P = 0.004$; order effect, $P = 0.07$; treatment *vs* order interaction, $P = 1.0$.

$P = 0.002$). Fewer patients had total or major control of vomiting on receiving LDMet during their second chemotherapy course (43% and 21% respectively), although there was no significant order effect or treatment *vs* order interaction.

Figures 4 and 5 show the results of the analysis of the diary cards completed during the 3-week period at home. The mean number of days during which vomiting occurred after the first course was 1.6 for group M/P and 1.95 for group P/M with neither treatment being superior. The percentage of patients experiencing delayed vomiting at any time after the first course was 55% (16/29) after LDMet and 50% (13/26) after LDPlac ($\chi^2_1 = 1.47$, $P>0.1$). Of the total patients (M/P and P/M) who experienced delayed emesis, vomiting continued after the first week in 21% (6/29), i.e 11% (6/55) of the total population who filled in diary cards. Figure 4 shows that patients experienced more nausea after the second course of treatment regardless of the anti-emetic treatment ($P = 0.0007$).

Table II shows patient preference. Choice was not influenced by the order in which the treatments were given and although more patients preferred LDMet this was not significant. Seven patients (11.5%) receiving metoclopramide had extrapyramidal reactions. One patient developed diabetes requiring subsequent oral hypoglycaemic treatment. One patient had severe oesophagitis and one reported nightmares. Patients receiving LDMet spent significantly more hours asleep than when receiving dexamethasone and lorazepam only. The mean number of hours asleep for all LDMet treatments was 11.7 (CI 10.6–12.8) while the mean number of hours spent asleep for all LDPlac treatments was 8.7 (CI 7.8–9.6, $P<0.001$).

**Figure 4** Mean number of days with vomiting (± 95% CI) following 1st and 2nd courses of chemotherapy for each group. Treatment effect, $P = 0.44$; treatment effect (1st period only), $P = 0.77$; order effect, $P = 0.36$; treatment *vs* order interaction, $P = 0.48$.

**Figure 5** Mean number of days with nausea (± 95% CI) following 1st and 2nd courses of chemotherapy for each group. Treatment effect, $P = 0.76$; treatment effect (1st period only), $P = 0.64$; order effect, $P = 0.0007$; treatment *vs* order interaction, $P = 0.79$.

**Table II** Preference of patients in each group for anti-emetic regimen

| Patient group | Preferred LDMet | Preferred LDPlac | Total (%) |
|---|---|---|---|
| Group M/P | 17 | 12 | 30 (54%) |
| Group P/M | 16 | 10 | 26 (46%) |
| Total | 33 (59%) | 23 (41%) | |

Treatment effect, $P = 0.59$; order effect, $P = 0.18$; treatment *vs* order interaction, $P = 0.71$.

### Discussion

This study shows that the combination of dexamethasone and lorazepam gives major emetic control in about 25% of patients. The regimen is simple to use and would probably be useful for patients receiving moderately emetic chemotherapy regimens without cisplatin. The addition of metoclopramide significantly improves emetic control. Forty-five per cent of patients had total and 67% major control when the triple drug combination was given on first exposure to chemotherapy, compared with 11% total and 25% major using dexamethasone and lorazepam only. The addition of metoclopramide also reduced the degree of nausea in the first 24 hours of treatment. These results are similar to those obtained in a double blind cross-over trial of high dose metoclopramide with or without dexamethasone reported by Allan *et al.* (1984). In 55 patients receiving 133 courses of cisplatin chemotherapy, total control of emesis was recorded in 43% of patients receiving metoclopramide plus dexamethasone, compared to 26% with metoclopramide plus placebo. There was also a significant reduction in the duration of nausea. In this study oral dexamethasone alone was as effective as oral dexamathasone plus oral metoclopramide in limiting nausea and vomiting during the 20 days between treatments. This is different to results recently reported by Kris *et al.* (1989). They described emesis in the four days post-chemotherapy in a group of patients who were all given the same anti-emetic treatment during their first 24 h in hospital and then randomised to receive oral placebo, oral dexamethasone or oral dexamethasone with oral metoclopramide. The oral dexamethasone with metoclopramide was significantly better than single agent dexamethasone or placebo during this assessment period (Kris *et al.*, 1989). This trial differs from the present trial in basic design, dose of metoclopramide and duration of assessment.

The incidence of extrapyramidal reactions in this present study was 11.5%. These have been described in as many as 20% of cases using various doses of metoclopramide (Strum *et al.*, 1982). In another study a much lower incidence of extrapyramidal reactions was reported, at all dosages and with all modes of administration (Kris *et al.*, 1983). In their large series of 452 patients, only 3% experienced extrapyramidal reactions, the incidence rising to 30% in patients under 30 years of age. The same group have reported that the addition of diphenhydramine and dexamethasone to metoclopramide both improved emetic control and suppressed the extrapyramidal side-effects (Kris *et al.*, 1985). Extrapyramidal side-effects in themselves did not appear to influence patients' choice in the present study. Choice was usually based on symptom control, general feeling of well being and time taken at home to recover. It is likely that the amnesic effect of lorazepam (Friedlander *et al.*, 1983) reduced patients' recall of their symptoms, reducing their perception of the differences between the two arms. The timing of the treatment to begin in the evening and carry on through the night masked any sedation effect of lorazepam.

There was a significant increase in the number of days of nausea experienced after all second courses regardless of the anti-emetic treatment. This may have been due to a real cumulative increase in this symptom with each course, or possibly a better understanding of symptoms by the patients with greater proficiency in filling in the diary card. Anticipatory nausea and vomiting did not occur during these first two courses of chemotherapy, nor was it expected, as a median of four to five courses are usually needed for the conditioned response to be established (Andrykowski, 1986; Fetting *et al.*, 1983).

Metoclopramide significantly contributes to anti-emetic control and will remain an important component of anti-emetic regimens until superior drugs become available. The combination of metoclopramide in high dose with dexamethasone, with or without lorazepam, represents the best available anti-emetic therapy for cisplatin containing regimens, and is the standard against which new agents or combinations should be tested.

### References

ALLAN, S.G., CORNBLEET, M.A., WARRINGTON, P.S., GOLLAND, I.M., LEONARD, R.C.F. & SMYTH, J.F. (1984). Dexamethasone and high dose metoclopramide: efficacy in controlling cisplatin induced nausea and vomiting. *Br. Med. J.*, **289**, 878.

ANDRYKOWSKI, M.A. (1986). Definitional issues in the study of anticipatory nausea in cancer chemotherapy. *J. Behav. Med.*, **9**, 33.

BAKER, J.J., LOKEY, J.L., PRICE, N.A., WINOKUR, S.H., BOWEN, J. & TAYLOR, A. (1979). Nabilone as an antiemetic. *N. Engl. J. Med.*, **301**, 728.

BOWCOCK, S.J., STOCKDALE, A.D., BOLTON, J.A.R., KANG, A.A. & RETSAS, S. (1984). Antiemetic prophylaxis with high dose metoclopramide or lorazepam in vomiting induced by chemotherapy. *Br. Med. J.*, **288**, 1879.

BREITBART, W. (1986). Tardive dyskinesia associated with high-dose intravenous metoclopramide. *N. Engl. J. Med.*, **315**, 518.

COATES, A., ABRAHAM,. S., KAYE, S.B., SOWERBUTTS, T., FREWIN, C., FOX, R.M. & TATTERSALL, M.H.N. (1983). On the receiving end–patient perception of the side-effects of cancer chemotherapy. *Eur. J. Cancer Clin. Oncol.* **19**, 203.

DRAPKIN, R.L., SOKOL, G.H., PALADINE, W.J., POLACKWICH, R. & LYMAN, G. (1982). The antiemetic effect and dose response of dexamethasone in patients receiving cisplatinum. *Proc. Am. Soc. Clin. Oncol.*, C 236.

FETTING, J.H., WILCOX, P.M., IWATA, B.A., CRISWELL, E.L., BOSMAJIAN, L.S. & SHEIDLER, V.R. (1983). Anticipatory nausea and vomiting in an ambulatory medical oncology population. *Cancer Treat. Rep.*, **67**, 1093.

FRIEDLANDER, M.C., SIMS, K. & KEARSLEY, J.H. (1983). Impairment of recall improves tolerance of cytotoxic chemotherapy. *Lancet*, **ii**, 686.

GRALLA, R.J., SQUILLANTE, A.E., STEELE, N., KELSEN, D.P. & YOUNG, C.W. (1980). Phase I intravenous trial of the antiemetic metoclopramide in patients receiving cis-platinum. *Proc. Am. Soc. Clin. Oncol.*, **21**, 350.

GRALLA, R.J., ITRI, L.M., PISKO, S.E. & 6 others (1981). Anti-emetic efficacy of high dose metoclopramide: randomised trial with placebo and prochlorperazine in patients with chemotherapy-induced nausea and vomiting. *N. Engl. J. Med.*, **305**, 905.

HILLS, M. & ARMITAGE, P. (1979). The two-period cross-over clinical trial. *Br. J. Clin. Pharmacol.*, **8**, 7.

KERR, D.J., GRAHAM, J., BLACKIE, R.G. & 4 others (1985). The relationship between steady state metoclopramide levels and control of emesis during treatment with cis-platinum. *Br. J. Clin. Pharmacol.*, **20**, 426.

KRIS, M.G., TYSON, L.B., GRALLA, R.J., CLARK, R.A., ALLEN, J.C. & REILLY, L.K. (1983). Extrapyramidal reactions with high dose metoclopramide. *N. Engl. J. Med.*, **309**, 433.

KRIS, M.G., GRALLA, R.J., TYSON, L.B. & 6 others (1985). Improved control of cisplatin induced emesis with high dose metoclopramide and with combinations of metoclopramide, dexamethasone and diphenhydramine. Results of consecutive trials in 255 patients. *Cancer*, **55**, 527.

KRIS, M.G., GRALLA, R.J., TYSON, L.B., CLARK, R.A., CIRRINCIONE, C. & GROSHEN, S. (1989). Controlling delayed vomiting: double-blind, randomized trial comparing placebo, dexamethasone alone, and metoclopramide plus dexamethasone in patients receiving cisplatin. *J. Clin. Oncol.*, **7**, 108.

MAHER, J. (1981). Intravenous lorazepam to prevent nausea and vomiting with cancer chemotherapy. *Lancet*, **i**, 91.

MEYER, B.R., LEWIN, M., DRAYER, D.E., PASMANTIER, M., LONSKI, L. & REIDENBERG, M.M. (1984). Optimising metoclopramide control of cisplatin-induced emesis. *Ann. Intern. Med.*, **100**, 393.

O'BRIEN, M.E.R. & CULLEN, M.H. (1988). Are we making progress in the management of cytotoxic drug-induced nausea and vomiting? *J. Clin. Pharmacol. Ther.*, **13**, 19.

STRUM, S.B., MCDERMED, J.E., OPFELL, R.W. & REICH, L.P. (1982). Intravenous metoclopramide: an effective anti-emetic in cancer chemotherapy. *J. Med. Assoc.*, **247**, 2683.

TAYLOR, W.B. & BATEMAN, D.N. (1983). High dose metoclopramide–preliminary pharmokinetic studies. *Br. J. Clin. Pharmacol.*, **16**, 341.

WARRINGTON, P.S., ALLAN, S.G., CORNBLEET, M.A., MACPHERSON, J.S., SMYTH, J.F. & LEONARD, R.C.F. (1986). Optimising antiemesis in cancer chemotherapy: efficacy of continous versus intermittent infusion of high dose metoclopramide in emesis induced by cisplatin. *Br. Med. J.*, **293**, 1334.